# Impact of coal mine dust exposure and cigarette smoking on lung disease in Appalachian coalminers

**DOI:** 10.1186/s12931-025-03260-3

**Published:** 2025-05-14

**Authors:** Rahul G. Sangani, Andrew J Ghio, Vishal Deepak, Javeria Anwar, Vinita Vaidya, Zalak Patel, Amirahwaty Abdullah

**Affiliations:** 1 Interstitial Lung Disease Clinic, Division of Pulmonary, Critical Care, and Sleep Medicine, West Virginia University School of Medicine, 1 Medical Center Dr, PO BOX 9166, Morgantown, WV 26506 USA; 2 US EPA, Chapel Hill, NC USA; 3 Department of Radiology, West Virginia University, Morgantown, WV USA

**Keywords:** Coal workers’ pneumoconiosis, High resolution CT chest, Cigarette smoking, Interstitial lung diseases, Fibrosis, Emphysema, Mortality

## Abstract

**Introduction:**

Interactions have been demonstrated between cigarette smoking (CS) and occupational exposures to several particles. This study tested the postulate that CS interacts with coal mine dust exposure to impact and change radiological and histological endpoints of coal mine dust lung disease.

**Methods:**

A retrospective evaluation of coalminers with a high-resolution computed tomography (HRCT) of the chest was conducted at West Virginia University Hospital (2015- 2022). There was a consensus review of both radiology and histology findings and their comparative analysis with a non-miner surgical resection cohort collected from thoracic oncology clinic.

**Results:**

The study cohort (n=556) was divided into groups: coal-/smoking- (8.3%), coal-/smoking+ (26.6%), coal+/smoking- (22.3%), and coal+/smoking+ (42.8%). Miners were older males with a median duration of coal mine work (CMW) of 30-years. Ever-smokers (66% of miner cohort and 76% of non-miner cohort) smoked 35 and 28 composite pack years (CPY) respectively, where miners had greater intensity of smoking (22 vs 18 cigarettes/day) compared to non-miners. On HRCT, 1/3^rd^ and 1/5^th^ of miners had simple and complicated coal workers’ pneumoconiosis (sCWP and cCWP), respectively. 35% of ever-smoking miners had radiologic patterns for probable usual interstitial pneumonitis, nonspecific interstitial pneumonitis, desquamative interstitial pneumonitis, and combined pulmonary fibrosis and emphysema. Radiologically, both coal-/smoking+ and coal+/smoking+ showed excessive emphysema (70-80%). Histologically, miners had more fibrosis (47% and 50% in coal+/smoking- and coal+/smoking+ vs. 11% and 28% in coal-/smoking- and coal-/smoking+). Never-smoking miners demonstrated more histological evidence of CWP than ever-smokers (60% and 27%); in addition, they had radiologic and histologic emphysema (30%), radiologic interstitial lung disease (ILD) (14.5%) and histologic evidence of fibrosis (47%). Ever-smokers demonstrated histologic emphysema more frequently (33% and 67% in coal+/smoking- and coal+/smoking+ vs. 24% and 72% in coal-/smoking- and coal-/smoking+). Logistic regression modeling showed the following associations: radiologic and histologic emphysema with CPY; histologic fibrosis, any ILD (not including RB-ILD), CPFE and anthracosis with both CPY and CMW; radiologic RB-ILD inclusive of small-opacities, cCWP with both CMW and silica; and sCWP and pulmonary artery dilation with CMW. Interestingly, CPY≥30 negatively correlated with radiologic cCWP and histologic CWP. Mortality was increased in smokers (14% and 29% in coal+/smoking- and coal+/smoking+ vs. 4% and 20% in coal-/smoking- and coal-/smoking+) with predictors being radiologic ILD, histologic CWP, and related co-morbid diseases including COPD, chronic kidney disease, and gastroesophageal reflux.

**Conclusion:**

CS demonstrated a major impact on miners’ health including changing radiologic and histologic endpoints of interstitial lung diseases and emphysema.

**Supplementary Information:**

The online version contains supplementary material available at 10.1186/s12931-025-03260-3.

## Introduction

In 2023, the active coalminer population in the United States numbered approximately 50,000, marking a decline from the 800,000 miners reported in the early 20^th^ century, with West Virginia leading the employment chart nationally [1]. A spectrum of coal mine dust lung disease (CMDLD) has been defined [2]. With a latency period of 20–30 years, the prevalence of coal workers’ pneumoconiosis (CWP) is estimated to affect 10–15% of miners. In addition to chronic bronchitis and/or emphysema, CMDLD also includes lesser-known entities of mixed dust pneumoconiosis and dust-related diffuse fibrosis (DDF) [2].

Relative to using the chest X-ray, there is an established greater sensitivity of high-resolution CT (HRCT) in detecting CMDLD [3]. The application of HRCT to diagnosing CMDLD varies globally [4–6]. While a semiquantitative approach to classifying the CT scan with pneumoconiosis can include using a standardized classification approach [7], HRCT has not been efficiently integrated into the routine health surveillance of miners. The continued risk of disease progression, even after coal mine dust exposure ceases, emphasizes a necessity for clinicians to maintain a heightened vigilance for miners'health [8].

Cigarette smoking (CS) remains a leading cause of preventable morbidity and mortality globally [9]. Central Appalachia contends with some of the highest rates of tobacco smoking nationwide with one in every fourth adults being a current smoker [10]. This habit is particularly prevalent among younger adults engaged in blue-collar industries like coal mining, resulting in a substantial burden of chronic respiratory diseases, diminished lung function, and decreased survival rates [11–15]. Historically, the impact of CS on coalminers’ health has garnered attention with documentation of more respiratory symptoms, increased emphysema/chronic bronchitis tissue changes, and greater airflow limitations on spirometry [16, 17]. Interest persists regarding the combined effects of smoking and coal mine dust exposure on lung tissue and function [7–12]. This study tested the postulate that CS interacts with coal mine dust exposure to impact and change radiological and histological endpoints of coal mine dust lung disease.

## Materials and methods

### Study design and settings

This single-center, retrospective cohort study was conducted at West Virginia University (WVU) and its affiliated hospitals, with approval from the WVU Institutional Review Board (ID#2210659320, November 18, 2022). The study cohort was defined using WVU electronic medical records system (i.e., EPIC) as patients: 1) having a medical evaluation between January 2015 to December 2022, 2) identifying their occupation as coal mining, 2) having good quality high resolution CT scan of the lung, and 4) adequate lung tissue for histopathology. For a comparative analysis, a non-miner cohort was selected from a group studied for suspicious nodules/masses [13]. Heavy smokers from this non-miner cohort (specifically quartiles 3rd and 4 th of smoking) were excluded to provide populations of miners and non-miners with equivalent smoking and to allow comparisons [13]. Such exclusion of heavy smokers did not result in any bias. Those individuals in the non-miner cohort were also required to have good quality high resolution CT (HRCT) scan of the lung, and adequate lung tissue for histopathology (acquired by video assisted or open thoracostomy). Neither the miner nor the non-miner cohorts included patients diagnosed to have lung cancer at the time of inclusion.

### Data collection

Detailed chart reviews were conducted to collect demographic information, comorbidities, and smoking history (categorized as never-smokers or ever-smokers, including current and former smokers). For ever-smokers, information on smoking duration, cigarettes smoked per day, resultant composite pack years (CPY), and years since cessation for former smokers were recorded. Occupational exposures to coal, silica, and asbestos were documented using electronic medical records.

### Radiographic Evaluation

HRCT chest scans with slice cuts ≤1.0 mm were analyzed by a team of pulmonologists and a radiologist. Consensus findings were recorded based on predefined case definitions derived from the medical literature. Radiographic CWP was categorized as simple (small opacity, ≤1 cm) or complicated (large opacities, >1 cm). Various interstitial lung diseases (ILDs), interstitial lung abnormalities (ILAs) patterns and emphysema subtypes were described (supplemental material). Pleural disease was categorized as thickness/plaques and rounded atelectasis. Lastly, dilated main pulmonary artery (PA) trunk was observed as an elevated the ratio of the main PA/Aorta (>0.9).

### Pathologic evaluation

For non-coal miners, all pathology specimens were from lobectomy. For coal miners, the pathology specimens were obtained from autopsy (n=8), surgical lung resection (lobectomy or segmentectomy, n=54), IR CT guided core-lung biopsy (n=10), and transbronchial lung biopsy (n=9). Consensus histopathologic findings reported from the lung tissue specimens were recorded retrospectively. Miners who had undergone surgical lung biopsy or autopsy during the study time frame were reported in addition to previously reported pathology findings from lung resection cohort as described by Sangani et al. [13]

### Statistical analysis

Means, medians, and standard deviations (SD) were used to summarize continuous variables while frequency distributions were used to describe categorical variables. Chi-square or Fisher exact tests were used to detect differences in categorical variables between the groups, while means of continuous variables were compared using one-way ANOVA. Tukey’s honest significant difference (HSD) calculator was employed to determine the difference between the individual groups. The Pearson correlation coefficient was used to measure the strength of a linear association between two continuous variables. Logistic regression analysis was used to determine associations of exposure (smoking, coal, silica, and asbestos) and various radiologic and histologic findings and to predict mortality of the coal miner cohort. The diagnoses or characteristics being studied includes emphysema (radiologic and histologic), interstitial lung disease (ILD, not including [or except] RB-ILD), respiratory bronchiolitis-ILD (RB-ILD), combine pulmonary fibrosis emphysema (CPFE) and histologic fibrosis), and coal workers pneumoconiosis (radiologic simple CWP, radiologic complicated CWP, histologic CWP, histologic anthracosis, dilated main pulmonary artery, and pleural plaque/thickening). Two-tailed tests of significance were employed (p<.05). Results of the investigation are described according to the STROBE statement. [19]

## Results

**Fig. 1:**
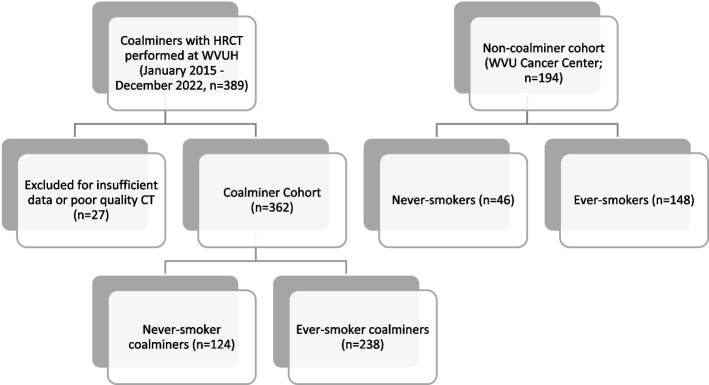
Study approach. Abbreviations: CWP= Coal Worker Pneumoconiosis, HRCT= High Resolution Computed Topography

Of 389 coalminers, 362 met the inclusion criteria with sufficient radiology available for evaluation (Fig. 1). Two-thirds of the coalminers were ever-smokers (Fig. 1). For a comparative analysis, non-miner patients from the lung resection cohort (for suspicious nodules/masses) were selected and divided into the groups of never smokers and ever-smokers (Fig. 1). Heavy smokers (third and fourth quartiles of CPY) from the lung resection (non-mining) cohort were excluded in an attempt to compare coalminers to a population with equivalent smoking. Demographics were significantly different between the groups (Table 1). The coalminers were older than the non-miners and predominantly male. Compared to never smokers, ever-smokers had a lower body mass index (BMI) in both groups of non-miners and miners. Miners worked for approximately 30 years with a higher proportion of never-smoking miners being underground. Silica and asbestos exposures were reported to be higher among the miners relative to non-miners.

**Table 1:** Baseline characteristics and mortality of cohort

**Variables** **Mean±SD or %**	**Non-coal miners** **(***n***=194)**		**Coal miners (***n***=362)**		*p***-value**
	**Never smokers** **(***n***=46)**	**Ever-smokers** **(***n***=148)**	**Never smokers** **(***n***=124)**	**Ever-smokers** **(***n***=238)**	
**Demographics**					
Age (years)	64.2±3.7	64.3±10.9	69.6±10.7	72.5±8.1	<.01^a^
Male, %	26.1	35.8	99.2	99.2	<.01^b^
BMI, mg/kg^2^	32.1±9.3	27.8±6.1	29.2 ± 5.7	26.9±6.0	<.01^c^
**Exposures**					
Coal, %	--	--	100	100	
Coal-mine work duration (years)	--	--	30.9±9.6	29.1±10.1	.15
Underground work, %	--	--	37.9	26.8	<.01^b^
Silica, %	8.7	7.4	20.9	19.7	<.01^b^
Asbestos, %	2.2	5.4	11.3	13.4	.02
**Smoking behavior**					
Current smoker, %	--	43.2	--	21.8	<.01^b^
Former smoker, %	--	56.8	--	78.2	<.01^b^
Years since quitting smoking	--	16.4±15.6	--	25.0±15.7	<.01^b^
Cigarettes per day	--	18.4±6.8	--	21.8 ±10.0	<.01^b^
Duration of smoking (years)	--	32.0±12.2	--	30.3 ± 16.2	.26
Composite pack years	--	27.9±12.0	--	34.6±25.8	<.01^b^
**Comorbidities, %**					
COPD	15.2	52.0	38.7	70.2	<.01^b^
Hypertension	63.0	68.2	65.3	70.6	.65
Hyperlipidemia	47.8	59.5	55.6	64.7	.11
CVA	4.3	10.1	10.5	8.8	.63
PAD	0	9.5	3.2	3.8	.01^b^
CHF	4.3	6.8	11.3	16.4	.01^b^
CAD	17.4	33.1	41.1	47.9	<.01^b^
Afib	8.7	10.8	15.3	22.7	<.01^b^
VTE	13.0	13.5	6.4	10.9	.28
Diabetes	30.4	27.0	30.6	28.6	.92
OSA	8.7	12.2	20.2	18.9	.10
CKD	8.7	6.8	7.3	11.3	.39
GERD	30.4	44.6	41.1	47.1	.19
Mood disorders	17.4	37.2	16.9	18.1	<.01^b^
Pain disorder	23.9	36.5	12.1	10.1	<.01^b^
Hypothyroidism	30.4	20.9	17.7	13.4	.02
Liver dysfunction	4.3	3.4	4.8	5.5	.82
Home O2 use	2.2	11.5	37.9	59.2	<.01^b^
**Mortality**	4.3	20.3	13.7	29.0	<.01^b^

*Afib* Atrial fibrillation, *CAD* coronary artery disease, *CHF* congestive heart failure, *CKD* chronic kidney disease, *COPD* chronic obstructive pulmonary diseases, *CVA* cerebro-vascular accidents, *GERD* gastro-esophageal reflux disease, *OSA* obstructive sleep apnea, *PAD* peripheral arterial disease, *VTE* venous thrombo-embolism

^a^significant difference noted between groups 1&3, 1&4, 2&3, 2&4, and 3&4

^b^Significant p-value with adjusted α=<.016 as per Bonferroni correction

^c^significant difference noted between groups 1&2, 1&3, 1&4 and 3&4

Smoking behavior showed significant differences between the non-miners and the miners (Table 1). Coalminers were more frequently former smokers with greater mean years since quitting the smoking habit. Miners smoked more cigarettes per day with greater composite pack years of smoking. However, the duration of smoking did not differ between non-miners and miners.

There was a substantial accumulation of comorbid conditions among smokers (Table 1). Ever-smoking miners had a statistically higher prevalence of COPD, coronary artery disease, atrial fibrillation, and congestive heart failure compared to other groups. Ever-smoking non-miners had more peripheral arterial disease, mood disorders, and pain disorders. Other common comorbid conditions included hypertension, hyperlipidemia, diabetes, and gastro-esophageal reflux disease (GERD) but there were no statistically significant differences between the groups. Home oxygen use was common among miners compared to the non-miner cohort.

**Fig. 2:**
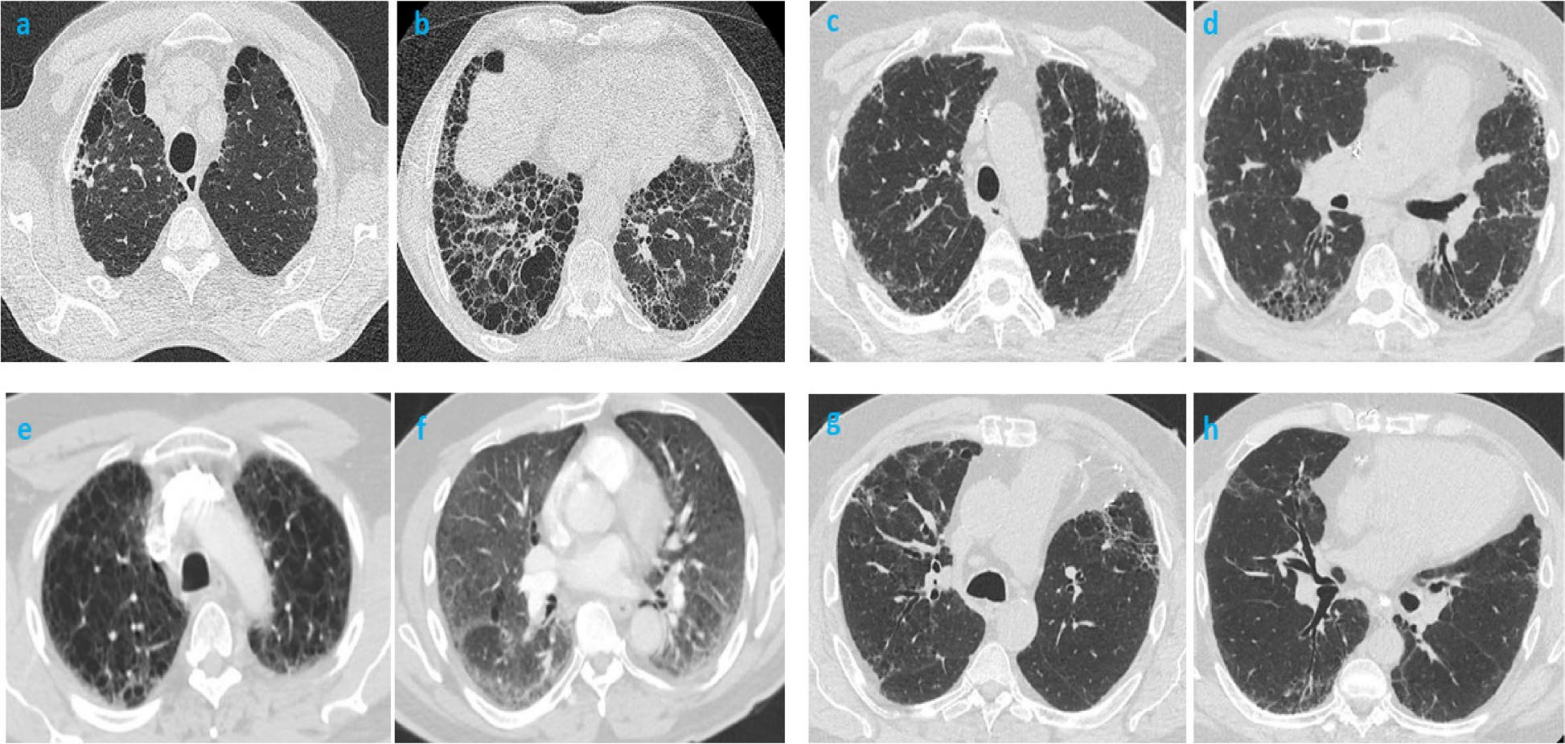
High resolution CT chest images with various interstitial lung disease patterns in coal miners. **a**, **b** HRCT chest images of 77-year-old-man with history of 23 years of coal mining work (both underground and surface, worked on every task with reported silica exposure) and former smoking of 40 pack years shows upper lobe predominant paraseptal and bullous emphysema and lower lobe predominant bibasilar extensive honeycomb changes with traction bronchiectasis, consistent with a pattern of combined pulmonary fibrosis emphysema (CPFE) and/or airspace enlargement with fibrosis (AEF). **c**, **d** HRCT chest images of a 69-year-old man with history of 42 years of mining (including 10 years of underground work), remote history of former smoking (7.5 pack years) shows apical to basal gradient of subpleural reticular changes, traction bronchiectasis and lower lobe predominant honeycomb changes, consistent with a pattern of usual interstitial pneumonia (UIP) who also underwent bilateral lung transplantations. Explant pathology confirmed UIP with anthracotic nodes and coal dust macules. **e**, **f** HRCT chest images of a 67-year-old-man with history of 25 years of coal mining work and current active smoker (1 pack per day for 50 years) shows upper lobe predominant emphysema (advanced centrilobular, paraseptal subtypes) and mid and lower zones predominant diffuse ground glass changes with reticular changes, likely suggesting of a pattern of desquamative interstitial pneumonia (DIP).**g**, **h** HRCT chest images of a 68-year-old long-wall continuous miner (for 43 years) and 50 pack years of cigarette smoking shows upper lobe predominant centrilobular emphysema, mediastinal calcified lymphadenopathy and an ILD pattern consistent with fibrotic nonspecific interstitial pneumonia (NSIP) characterized by non-apical to basal gradient, scattered ground glass opacities and traction bronchiectasis

HRCT chest findings of the cohort are described in Table 2. One-third of miners had sCWP observed on the HRCT scan whereas 22.7% had cCWP with a trend towards never-smoking miners (28.2% vs. 19.7%, p=.067). Their radiologic characteristics according to cigarette smoke exposure are presented (Supplemental Table T1). Miners with ever-smoking showed a significant increase in observations of any ILD (not including respiratory bronchiolitis (RB)-ILD), probable usual interstitial pneumonia (UIP), nonspecific interstitial pneumonia (NSIP), RB-ILD (inclusive of small opacities (≤1 cm) of sCWP mimicking RB-ILD), desquamative interstitial pneumonitis (DIP), and combined pulmonary fibrosis emphysema (CPFE) patterns. Approximately 30–40% of both cohorts had ILA patterns. Among them, centrilobular ground glass opacities (CL-GGO) were noted more commonly in non-miners whereas, there was a trend towards significance for mixed CL-GGO and subpleural reticulation subtype in ever-smoking miners. Isolated traction bronchiectasis/bronchiolectasis seen in about 20% of cohort with trend towards ever-smokers. Compared to never smokers, ever-smokers had an excessive prevalence of radiologic emphysema with centrilobular being the most common subtype. There was a smaller impact of coal mine dust exposure on paraseptal, panacinar and bullous emphysema and concomitant smoking in miners increased the observed percentages of these subtypes. For miners, cicatricial emphysema was characterized without significant difference between never- and ever-smokers. Pleural plaques/thickening was seen more commonly in miners coinciding with their asbestos exposure. Approximately one-fifth of cohort had dilated main pulmonary artery without significant difference between the groups. HRCT images from the representative cases are displayed in Fig. 2.

**Table 2:** Radiologic findings of cohort

	**Non-coal miners** **(***n***=194)**		**Coal miners (n=362)**		*p***-value**
	**Never smokers** **(***n***=46)**	**Ever-smokers** **(***n***=148)**	**Never smokers** **(***n***=124)**	**Ever-smokers** **(***n***=238)**	
**ILD patterns**^**a**^					
Simple CWP, %	0	0	32.3	34.5	.67
Complicated CWP, %	0	0	28.2	19.7	.07
Any ILD, not including RB-ILD, %	0	16.2	14.5	34.9	<.01^a^
UIP %	0	0.7	2.4	2.5	.42
Probable UIP %	0	1.4	0.8	5.0	.03
NSIP %	0	0.7	4.8	6.7	.01
RB-ILD (including small opacities) %	0	3.4	25.0	12.3	<.01^a^
DIP %	0	2.0	0	8.0	<.01^a^
CPFE %	0	2.0	0.8	16.0	<.01^a^
Organizing pneumonia %	0	2.7	0.8	0.8	.43
Asbestosis %	0	0	2.4	0.8	.17
Other/unclassifiable %	0	4.1	2.4	1.7	.25
**ILA patterns**	36.9	43.2	29.8	34.0	.12
CL-GGO %	13.0	15.5	8.9	6.3	.02
SPR %	17.4	17.6	8.1	11.8	.09
Mixed CL-GGO+SPR %	10.9	11.5	17.7	21.0	.07
Non-emphysematous cysts %	10.9	7.4	8.1	8.4	.91
Traction bronchiectasis/bronchiolectasis %	21.7	26.3	13.7	18.5	.06
**Any Emphysema**	13.0	87.8	29.0	77.7	<.01^a^
Centrilobular %	10.9	79.7	16.9	71.0	<.01^a^
Paraseptal %	2.2	34.5	3.2	53.4	<.01^a^
Panacinar %	0	8.1	0.8	9.7	<.01^a^
Bullous %	0	4.1	2.4	21.4	<.01^a^
Cicatricial %	-	-	18.6	15.1	.40
Pleural plaque/thickening %	0	5.4	14.5	16.8	<.01^a^
Dilated main pulmonary artery (ratio PA/Aorta >0.9), %	15.2	18.9	23.4	24.8	.35

*CL* centrilobular, *CPFE* combined pulmonary fibrosis emphysema, *CWP* Coal-workers’ pneumoconiosis, *DIP* Desquamative interstitial pneumonia, *GGO* ground glass opacity, *ILD* interstitial lung disease; *NSIP* nonspecific interstitial pneumonia, *RB* respiratory bronchiolitis, *SPR* subpleural reticular changes, *UIP* usual interstitial pneumonia

^a^Significant *p*-value with adjusted α=<.016 as per Bonferroni correction

Lung pathology findings are available for all non-miners and 22.4% of coalminers (in 12.1% and 27.7% never-smoking and ever-smoking miners, respectively) and are described (Table 3). Histologic evidence of CWP was observed twice as frequently among never-smoking miners compared to ever-smoking miners. Half of miners’ available lung tissue showed fibrotic changes, approximately doubling the frequency seen in non-mining smokers. Except for a trend demonstrating an RB pattern among ever-smokers, no other pathological pattern showed significant differences between groups. A higher proportion of miners (67.9%) had anthracosis compared to non-miners (45.7%). Histologic emphysema was found more frequently among ever-smokers (Table 3). One-third of never-smoking miners had histologic evidence of emphysema. Chronic inflammation was observed more frequently in ever-smoking miners compared to other groups.

**Table 3:** Pathologic findings of cohort

**Variables** **Mean±SD or %**	**Non-coal miners**		**Coal miners**		*p***-value**
**Lung tissue available**^**,a,b**^	**Never smokers** **(***n***=46)**	**Ever-smokers** **(***n***=148)**	**Never smokers** **(***n***=15)**	**Ever-smokers** **(***n***=66)**	
Coal-workers’ pneumoconiosis, %	0	0	60.0	27.3	.04
Fibrosis, %	10.9	27.7	46.7	50.0	<.01^c^
Usual interstitial pneumonia, %	0	2.0	0	4.5	.37
Organizing pneumonia, %	6.5	7.4	6.7	7.6	.99
Peribronchiolar metaplasia, %	2.2	10.1	6.7	4.5	.23
Respiratory bronchiolitis, %	0	11.5	0	7.6	.05
Desquamative interstitial pneumonia, %	0	5.4	0	7.6	.22
Anthracosis, %	10.9	56.8	80.0	65.2	<.01
Emphysema, %	23.9	71.6	33.3	66.7	<.01^c^
Granuloma morphology:					
Necrotizing, %	8.7	1.4	0	3.0	.07
Non-necrotizing (sarcoid-like), %	6.5	4.7	6.7	7.6	.86
Calcified, %	0	4.7	0	3.0	.57
Chronic inflammation, %	4.4	1.4	6.7	13.6	<.01^c^

^a^Individuals may have more than one pathologic finding

^b^CWP morphology patterns reported on pathology included coal macule/nodule (*n*=18), silicotic nodule (*n*=6), and PMF (*n*=8)

^c^Significant *p*-value with adjusted α=<.016 as per Bonferroni correction

A logistic regression model showed significant associations between 1) CPY≥30 years, coal mine work (CMW) duration ≥30 years, and silica and asbestos exposure with 2) radiologic and histologic findings (Table 4). A radiologic ILD (not including RB-ILD) pattern was related to CPY≥30, CMW≥30 years, and silica exposure (OR 2.03 [1.15–3.58], p=.014). A similar association was seen for RB-ILD pattern (inclusive of small opacities of sCWP) except for CPY≥30. CPFE pattern, histologic fibrosis and anthracosis showed strong associations with both CPY≥30 and CMW≥30 years. While CMW≥30 years predicted radiologic sCWP and cCWP, CPY≥30 demonstrated an inverse relationship with PMF lesions. Like radiologic CWP, histologic CWP was inversely correlated with CPY≥30 and predicted by CMW≥30 years. Smoking (CPY≥30) predicted both radiologic emphysema (OR 11.06 [6.64–18.40], p=0) and histologic emphysema. The dilatation of the main pulmonary artery trunk showed a positive relationship with CMW≥30 years. These associations of ILA patterns, histologic DIP, chronic inflammation, and emphysema subtypes (centrilobular, paraseptal, panacinar, bullous and cicatricial) with model variables are provided (Supplemental Table T2).

**Table 4:** Multivariate logistic regression model predicting significant associations between radiologic and histologic findings and exposures in the coal miner cohort

	**Composite smoking pack ≥30 years**			**Coal mine work duration ≥30 years**			**Silica exposure**			**Asbestos exposure**		
	**OR**	**95% CI**	*p***-value**	**OR**	**95% CI**	*p***-value**	**OR**	**95% CI**	*p***-value**	**OR**	**95% CI**	*p***-value**
**Emphysema**												
Radiologic	11.06	6.64–18.40	<.001									
Histologic	3.80	2.20–6.56	<.001									
**ILDs**												
Any ILD (not including RB-ILD)	3.67	2.38–5.65	<.001	1.73	1.12–2.67	.01	2.03	1.15–3.58	.01			
RB-ILD (including small opacities disease)	0.33	0.16–0.68	.002	5.99	3.15–11.41	<.001	3.18	1.61–6.29	<.001			
CPFE	10.4	4.49–24.34	<.001	2.44	1.24–4.78	.009						
Histologic fibrosis	2.62	1.48–4.61	<.001	3.27	1.65–6.48	<.001						
**CWP**												
Radiologic simple CWP				3.51	2.15–5.73	0						
Radiologic complicated CWP	0.52	0.29–0.90	.02	5.49	3.19–9.41	0	3.53	1.91–6.51	<.001			
Histologic CWP	0.21	0.06–0.69	.01	19.30	6.45–57.69	0						
Histologic anthracosis	2.45	1.48–4.06	<.001	1.99	1.01–3.96	.05						
Dilated main pulmonary artery (CT ratio PA/Ao> 0.90)				1.58	1.05–2.38	.03						
Pleural plaque/thickening (radiologic)										4.98	2.63–9.41	<.001

*CWP* coal workers’ pneumonoconiosis, *CPFE* combined pulmonary fibrosis with emphysema, *ILD* interstitial lung disease, *OR* odds ratio (unadjusted), *PA/Ao* ratio of main pulmonary artery trunk and ascending aorta diameter, *RB-ILD* respiratory bronchiolitis-ILD

One-fifth (21.2%) of cohort patients died within the timeframe of the study. Mortality was significantly increased among the ever-smokers and never smoking miners (Table 1). Univariate analysis identified many predictors of mortality in the cohort including coal mine work and smoking behavior (Supplemental Table T3). Based on these, a multivariate logistic regression model was developed (Table 5). Baseline comorbid conditions of underweight, COPD, chronic kidney disease (CKD), GERD and hypoxemic respiratory failure with home O_2_ use were significantly associated with mortality. Both ILD patterns (not including RB-ILD) and histologic CWP correlated positively with mortality.

**Table 5:** Multivariate logistic regression model of significant (p<.05) mortality predictors

	**OR**	**95% CI**
Body mass index (BMI), kg/m^2^	0.96	0.92–0.99
Clinical COPD diagnosis	1.94	1.18–3.19
Chronic Kidney Disease	3.24	1.64–6.41
GERD	1.67	1.07–2.63
Home Oxygen use	2.12	1.32–3.42
Any ILD (not including RB-ILD)	1.96	1.19–3.21
Histologic CWP	3.09	1.25–7.62

*COPD* chronic obstructive pulmonary disease, *CWP* coal workers’ pneumoconiosis, *GERD* gastro-esophageal reflux disease, *ILD* interstitial lung disease, *RB-ILD* respiratory bronchiolitis-ILD

## Discussion

The detailed assessment of smoking habits in this cohort provides an opportunity for an understanding of its comprehensive impact. The prevalence of ever-smoking among miners in this study population is comparable to that observed in other cohorts of CWP in this region [20]. A historic cohort of autopsies (1957-1978) examining the effects of coal mine dust and smoking on miners’ lung disease, a comparable CPY was estimated although smoking history was unavailable for 30% of the cohort [21]. This pattern of smoking exposure affected miners from the early and the mid-20 th century, reflecting the era's pervasive smoking culture. As a result of the prohibition of smoking in underground mines, it is likely that miners had higher smoking intensity when not working underground rather than extended durations of smoking, which did not significantly differ from non-miner smoker controls. It is reasonable to assume that our mining cohort accrued a significant particle burden in their lungs over time. Unsurprisingly, such tobacco smoke exposure correlated with a proportional increase in comorbidities including COPD, cardiovascular diseases, malnutrition (reflected in lower BMI), and hypoxemic respiratory failure among smokers, including miners. [22, 23]

The latest classification of ILDs includes CWP resulting from occupational exposures [24]. Through the application of standard interpretation techniques for HRCT, it has been revealed that approximately one-third of smoking miners and 15% of non-smoking miners exhibit non-CWP ILD patterns (not including RB-ILD). Previous post-mortem examinations have demonstrated interstitial fibrosis in 15–20% of miners in the region [25]. Both excessive smoking (with CPY exceeding 30) and cumulative coal mine dust exposure were strongly correlated with radiologic ILD patterns (excluding RB), CPFE pattern, and histologic fibrosis. A review supported an association between non-quartz coal mine dust and ILD risk though smoking has often been overlooked as a confounding factor [26]. In our cohort, ever-smoking miners showed association with diverse array of ILD patterns (including CPFE, DIP, probable UIP, and NSIP), all are known to correlate with smoking [27–30]. Past investigation supports an association between coal mine dust exposure and conditions such as CPFE, chronic interstitial pneumonia with honeycombing, and DIP [27, 31–34]. The phenomenon of an interstitial fibrosis from coal mine dust, designated DDF, has been observed in approximately 30% of autopsied miners from Wales [27], with a clinical trajectory demonstrating deterioration comparable to IPF. Animal studies have supported coal dust-induced oxidative stress, downstream activation of transcription factors leading to chronic inflammation and fibrosis, identical to smoking-induced lung damage [35]. In addition to particle size, composition of coal mine dust, including quartz and iron content, have been implicated in the development of interstitial fibrosis. [36–39]

Simple CWP can mimic RB-ILD due to the centrilobular deposition of dust, macrophages, and connective tissue and differentiating it from smoking-related lung injury can sometimes present a challenge. Non-smoking miners show a higher prevalence of RB-ILD, characterized by small opacities consistent with sCWP [4]. Cumulative coal mine work duration showed strong associations with the entire spectrum of CWP including radiologic sCWP, cCWP, RB-ILD patterns (both smoking plus coal-associated small opacities) as well as histologic anthracosis and histologic CWP. In addition, silica exposure strongly predicted radiologic RB-ILD and cCWP. The complex interaction of concurrent smoking on CWP revealed possible protective effects in the logistic regression model. A negative interaction of coal content of lungs (histologic) and smoking was observed [40]. This is comparable to the described effects of smoking on decreasing sarcoidosis and hypersensitivity pneumonitis [41]. The chronic inhalation of a particle e.g., cigarette smoke particle can accelerate a release of monocytes from the bone marrow which is followed by recruitment into the lung, and differentiation to macrophages (accounting for increased numbers), allowing their participation in critical clearance from the distal respiratory tract [42, 43]. With chronic particle overload (observed with higher exposures), there is an impairment of particle clearance mediated by macrophages which normally regulate the process [44]. Accordingly, with smoking, coal mine dust may not be successfully translocated to the respiratory/terminal bronchioles to contribute to the development of the macule/nodule. However, the coal mine dust would then accumulate in more distant sites and may contribute to alternative types of inflammation/fibrosis (e.g. DIP, DDF, organizing pneumonia, NSIP, and UIP). Approximately one-third of the cohort exhibited subclinical ILAs, commonly associated with smoking but were also present in non-smoking miners. Fibrotic ILAs have been linked to disease progression and unfavorable outcomes [45, 46]. Intriguingly, irregular opacities observed in smoking miners had been histologically linked to emphysema and interstitial fibrosis [47–49]. The ubiquitous presence of irregular opacities on chest X-rays, irrespective of exposure profile, has long posed a challenge in epidemiological studies. It is imperative to explore whether there exists a correlation between incidentally detected ILAs on HRCT and irregular opacities on chest X-rays in miners.

Localized emphysema, a distinctive feature of CWP, manifests as an expansion of second- and third-order respiratory bronchioles, accompanied by the infiltration of coal mine dust-laden macrophages into the bronchiolar walls, minimal collagen deposition and a reduction in smooth muscle thickness. In this study, smoking miners displayed notably elevated rates of both radiologic and histologic emphysema. In addition, approximately one-third of non-smoking miners also exhibited emphysema. Investigation of autopsied tissue supported additive effects of coal mine dust and smoking to the development of emphysema [16, 21, 50]. Smoking bituminous coalminers demonstrated higher rates of emphysema and chronic bronchiolitis (bronchiolar goblet cells hyperplasia) compared to non-smoking miners [17]. The extent of emphysema in miners has been linked to lung coal content, age, smoking, and the severity of pneumoconiosis, whereas in lifelong non-smokers, emphysema was strongly related to coal content and age [40, 51]. Identical to our findings, centrilobular emphysema emerged as the most prevalent subtype among miners. Smoking miners exhibited a higher prevalence of centriacinar emphysema compared to non-mining controls with similar smoking histories [52, 53]. The occurrence of centrilobular emphysema was associated with greater coal mine dust exposure, particularly in cases of PMF [54]. Paraseptal, bullous, and pan-acinar emphysema are common subtypes observed among smoking miners, a trend corroborated by quantitative CT evaluations conducted in German miners (except bullous type) [55]. Logistic regression analyses in this cohort identified CPY as the sole predictor of both radiologic and histologic emphysema. Similarly, white smoking miners from South Africa demonstrated comparable predictive trends. [56]

Increased mortality has been observed among miners exposed to respirable coal mine and silica dust [57]. Concurrent smoking, complicated pneumoconiosis, and specific coal rank regions can modify this risk [21, 58, 59]. A historical analysis of Appalachian miners revealed that cigarette smoking and airway obstruction contributed to excess mortality [60]. Our study identified increased mortality for one in three miners who ever smoked. Mortality within our cohort was associated with various comorbid conditions, radiologic ILD (not including RB-ILD), and histologic CWP. Lower BMI is well-known indicator of poor prognosis in COPD [61] and malnutrition more frequently afflicted patients with combined COPD and CWP [5]. Smoking has been repeatedly identified as a risk factor for GERD [62], possibly explaining a higher burden among smoking miners. Similarly, Appalachian coalminers exhibited increased mortality rates associated with chronic heart, respiratory and kidney diseases, possibly reflecting smoking [63]. Unsurprisingly, a higher burden of COPD, ILD and PMF contributed towards a need for home oxygen in two-third of smoking miners in our cohort.

There are limitations of the retrospective analysis used in our study. The control group consisted of patients undergoing surgical lung resection for suspicious lung nodules/masses and had higher prevalence of overall smoking and lung cancer [13]. This contrasts only one-fifth of the miners having histologic findings available. Study findings describe the characteristics of miners who live in central Appalachia and extrapolation to other mining populations may be limited. Furthermore, the disproportionately male-specific nature of mining employment limits the understanding for effects of coal mine dust among women. Strengths of this investigation includes large sample size with detailed description of participants’ exposure profiles, clinical features, and systematic assessment of CT chest findings, thereby enhancing the robustness of study findings.

## Conclusion

Distinct and excessive smoking habits are prevalent in the central Appalachian miners who are already inhaling higher concentrations of coal mine dust relative to other regions [64]. Consequently, deleterious effects of smoking manifested with greater accumulation of radiologic and histologic evidence of ILDs, emphysema, and overall poor outcome among smoking miners. Our findings support an interaction between coal mine dust and smoking to impact non-CWP fibrosis identified both radiologically and histologically. The disproportionate burden of chronic respiratory diseases, coupled with the inadequate incorporation of advanced imaging exacerbates existing disparities within this already marginalized population. There is an urgent need to transition towards routine use of CT chest evaluation complementing the existing B-reading framework in assessing coal mine dust lung disease [65, 66]. Mobile lung cancer screening units have been successfully implemented in rural Appalachian settings. Therefore, integration of similar platforms may offer a convenient option for the region [67]. Finally, surveillance of miners must include assessment of concomitant smoking and early appropriate referral to cessation programs.

## Supplementary Information

Supplementary file 1.

Supplementary file 2.

## Data Availability

No datasets were generated or analysed during the current study.
