# Supplementary material for: Impact of coal mine dust exposure and cigarette smoking on lung disease in Appalachian coalminers
Source: Respir Res. 2025 May 14;26:184. doi: 10.1186/s12931-025-03260-3 (PMC12079898; doi:10.1186/s12931-025-03260-3)
Supplement: Supplementary file 2 [file 12931_2025_3260_MOESM2_ESM.docx]

**Supplementary material:**

**Methods:**

Details recorded for simple CWP (sCWP) included size (≤3 mm or >3-10 mm), upper vs. lower lobe location, calcification, laterality, and distribution (centrilobular, perilymphatic, or random). Complicated CWP (cCWP) descriptions encompassed size, laterality, location (central vs. peripheral), lobar distribution, fissural involvement, and calcification patterns (diffuse, punctate, and eggshell). Involvement of mediastinal and/or hilar lymph nodes, along with their calcification patterns (diffuse, punctate, eggshell), was noted. Interstitial lung diseases (ILDs) were classified according to established criteria; these included usual interstitial pneumonia (UIP), probable UIP, non-specific interstitial pneumonia (NSIP), respiratory bronchiolitis (RB)-ILD, pulmonary Langerhans cells histiocytosis (pLCH), desquamative interstitial pneumonitis (DIP), combined pulmonary fibrosis emphysema (CPFE), organizing pneumonia, asbestosis, and unclassifiable patterns.^1-4^ Interstitial lung abnormalities (ILAs) were identified based on predefined definitions, encompassing patterns such as centrilobular ground glass opacities (GGO), sub-pleural reticulations, non-emphysematous cysts, architectural distortion with isolated traction bronchiectasis/bronchiolectasis, and isolated honeycombing involving at least 5% of a lung zone in a non-dependent fashion.^5,6^ Emphysema and its various subtypes, including centrilobular, paraseptal, panacinar, and bullous, were documented. Emphysema immediately adjacent to CWP lesions was termed para-cicatricial. Attention was paid to describe ILDs and ILAs in non-CWP regions.

**References**:

1. American Thoracic Society; European Respiratory Society. American Thoracic Society/European Respiratory Society International Multidisciplinary Consensus Classification of the Idiopathic Interstitial Pneumonias. This joint statement of the American Thoracic Society (ATS), and the European Respiratory Society (ERS) was adopted by the ATS board of directors, June 2001 and by the ERS Executive Committee, June 2001. Am J Respir Crit Care Med. 2002 Jan 15;165(2):277-304. doi: 10.1164/ajrccm.165.2.ats01.

2. Mueller-Mang C, Grosse C, Schmid K, Stiebellehner L, Bankier AA. What every radiologist should know about idiopathic interstitial pneumonias. Radiographics. 2007 May-Jun;27(3):595-615. doi: 10.1148/rg.273065130.

3. Travis WD, Costabel U, Hansell DM, King TE Jr, Lynch DA, Nicholson AG et al. ATS/ERS Committee on Idiopathic Interstitial Pneumonias. An official American Thoracic Society/European Respiratory Society statement: Update of the international multidisciplinary classification of the idiopathic interstitial pneumonias. Am J Respir Crit Care Med. 2013 Sep 15;188(6):733-48. doi: 10.1164/rccm.201308-1483ST.

4. Cottin V, Selman M, Inoue Y, Wong AW, Corte TJ, Flaherty KR et al. Syndrome of Combined Pulmonary Fibrosis and Emphysema: An Official ATS/ERS/JRS/ALAT Research Statement. Am J Respir Crit Care Med. 2022 Aug 15;206(4):e7-e41. doi: 10.1164/rccm.202206-1041ST.

5. Hata A, Schiebler ML, Lynch DA, Hatabu H. Interstitial Lung Abnormalities: State of the Art. Radiology. 2021 Oct;301(1):19-34. doi: 10.1148/radiol.2021204367.

6. Hatabu H, Hunninghake GM, Richeldi L, Brown KK, Wells AU, Remy-Jardin M et al. Interstitial lung abnormalities detected incidentally on CT: a Position Paper from the Fleischner Society. Lancet Respir Med. 2020 Jul;8(7):726-737. doi: 10.1016/S2213-2600(20)30168-5.

**Supplemental Table T1: Radiologic features of coal workers’ pneumoconiosis as per the cigarette smoke exposure**

| Variables, % | Coal miners (n=362) | | p-value |
| --- | --- | --- | --- |
|  | **Never smokers**  **(n=124, 34.3%)** | **Ever-smokers**  **(n=238, 65.7%)** |  |
| Simple CWP,% | 32.3 | 34.5 | .67 |
| Small opacities (<=1 cm) features |  |  |  |
| Unilateral | 2.4 | 1.7 | .63 |
| Bilateral | 29.8 | 32.8 | .57 |
| Upper lobe predominant | 21.0 | 26.0 | .28 |
| Lower lobe predominant | 0 | 1.7 | .30 |
| Diffuse involvement | 9.7 | 6.7 | .41 |
| Calcified | 8.1 | 10.9 | .46 |
| Size: <=3 mm | 24.2 | 25.2 | .89 |
| Size: >3-10 mm | 8.1 | 9.7 | .70 |
| Distribution patterns of small opacities: |  |  |  |
| Centrilobular | 14.5 | 16.8 | .65 |
| Peri-lymphatic | 4.8 | 8.0 | .38 |
| Mixed centrilobular and peri-lymphatic | 11.3 | 6.7 | .16 |
| Random | 1.6 | 3.8 | .34 |
| Complicated CWP, % | 28.2 | 19.7 | .07 |
| PMF lesions (>=1 cm) features |  |  |  |
| Unilateral | 7.3 | 4.2 | .22 |
| Bilateral symmetric | 12.9 | 8.0 | .14 |
| Bilateral asymmetric | 8.9 | 7.6 | .67 |
| Central | 21.0 | 10.9 | .01 |
| Peripheral | 11.3 | 10.1 | .72 |
| Lobar distribution: |  |  |  |
| Right upper lobe | 26.6 | 18.1 | .07 |
| Right middle lobe | 11.3 | 6.3 | .11 |
| Right lower lobe | 8.1 | 5.0 | .25 |
| Left upper lobe | 19.3 | 16.4 | .47 |
| Left lower lobe | 4.8 | 4.2 | .79 |
| Calcification, any | 17.7 | 12.2 | .15 |
| Diffuse calcification | 0 | 1.7 | .30 |
| Punctate calcification | 13.7 | 10.5 | .39 |
| Eggshell calcification | 2.4 | 0 | .04 |
| Fissure involvement | 20.2 | 11.8 | .04 |

**Supplemental Table T2: Multivariate logistic regression model showing significant associations for additional radiologic and histologic findings of coal miner cohort**

|  | Composite smoking pack ≥30 years | | | Coal mine work duration ≥30 years | | | Silica exposure | | | Asbestos exposure | | | |
| --- | --- | --- | --- | --- | --- | --- | --- | --- | --- | --- | --- | --- | --- |
|  | **OR** | **95% CI** | **p-value** | **OR** | **95% CI** | **p-value** | **OR** | **95% CI** | **p-value** | **OR** | | **95% CI** | **p-value** |
| Radiologic emphysema subtypes | | | | | | | | | | | | | |
| a.Centrilobular | 9.06 | 5.88 – 13.95 | 0 |  |  |  |  |  |  |  |  | |  |
| b.Paraseptal | 6.61 | 4.46 – 9.81 | 0 |  |  |  |  |  |  |  |  | |  |
| c.Panacinar | 4.81 | 2.27 – 10.17 | 0 |  |  |  |  |  |  |  |  | |  |
| d.Bullous | 5.64 | 3.05 – 10.41 | 0 |  |  |  | 2.83 | 1.40 – 5.74 | .003 |  |  | |  |
| e.Cicatricial | n.s. |  |  | 2.20 | 1.18 – 4.11 | .013 | 2.27 | 1.18 – 4.37 | .013 |  |  | |  |
| Radiologic ILA pattern | | | | | | | | | | | | | |
| a. CL-GGO ILA |  |  |  |  |  |  |  |  |  | 2.35 | 1.01 – 5.45 | | .046 |
| b. SPR ILA |  |  |  | 0.58 | 0.33 – 1.03 | .060* |  |  |  |  |  | |  |
| Additional histologic findings | | | | | | | | | | | | | |
| DIP | 7.59 | 1.64 – 35.02 | .009 |  |  |  |  |  |  |  |  | |  |
| Chronic inflammation |  |  |  | 8.65 | 2.79 – 26.80 | <.001 |  |  |  |  |  | |  |

Radiologic ILAs (inclusive of all patterns) and histologic RB and granulomatous inflammation did not identify any significant associations.

*trend towards significance

**Supplemental Table T3: Univariate analysis of significant (p<.05) mortality predictors of the group**

| Variables  Mean±SD or % | Alive  (n=438, 78.8%) | Dead  (n=118, 21.2%) |
| --- | --- | --- |
| Age (years) | 68.06±10.72 | 72.57±9.56 |
| BMI, kg/m^2^ | 28.60±6.36 | 26.18±6.39 |
| Male, % | 73.9 | 84.7 |
| Comorbidities, % |  |  |
| COPD | 48.4 | 73.7 |
| Hyperlipidemia | 57.7 | 67.8 |
| Cerebrovascular accident | 7.5 | 15.2 |
| Congestive heart failure | 10.1 | 17.8 |
| Coronary artery disease | 37.2 | 50.0 |
| Atrial fibrillation | 15.1 | 22.9 |
| Diabetes | 28.1 | 31.4 |
| Chronic kidney disease | 6.6 | 17.8 |
| Gastro-esophageal acid reflux | 40.4 | 55.9 |
| Squamous cell lung carcinoma | 6.8 | 12.7 |
| Home O_2_ use | 31.3 | 60.2 |
|  |  |  |
| Exposures |  |  |
| Coal mine work ≥30 years, % | 63.0 | 72.9 |
| Ever-smokers, % | 65.7 | 83.9 |
| Ex-smokers, % | 44.7 | 63.6 |
| Cigarettes smoke per day | 19.60±8.40 | 23.00±10.60 |
| Composite pack years | 30.30±19.82 | 36.69±25.54 |
| CPY≥30 years, % | 34.9 | 53.4 |
|  |  |  |
| Radiology findings, % |  |  |
| Any emphysema | 60.7 | 77.1 |
| Centrilobular emphysema | 52.5 | 69.5 |
| Paraseptal emphysema | 29.0 | 47.5 |
| Bullous emphysema | 8.9 | 17.8 |
| Any ILD | 27.6 | 46.6 |
| ILD without RB-ILD | 17.1 | 39.0 |
| UIP | 1.1 | 4.2 |
| DIP | 3.0 | 7.6 |
| CPFE | 5.0 | 16.9 |
| PA/Aorta diameter >0.9 | 19.9 | 30.5 |
|  |  |  |
| Pathology findings, % |  |  |
| Fibrosis | 25.6 | 52.5 |
| CWP | 6.8 | 21.3 |
